# Re: Insufficient conclusions regarding the association between overactive bladder symptoms and degree of dementia

**DOI:** 10.1590/S1677-5538.IBJU.2017.0171

**Published:** 2017

**Authors:** Jae Heon Kim

**Affiliations:** 1 Department of Urology, Soonchunhyang University Hospital, Soonchunhyang University Medical College, Seoul, Korea

*To the editor,*

Recently, Jung et al. (1) reported a remarkable study on a possible association between the symptoms of overactive bladder (OAB) and the degree of dementia. Considering the paucity of relevant studies on this issue, this study has value. However, several issues could be upgraded by the authors to contribute to a better understanding of the subject.

First, there are several pitfalls in the authors’ methodology, including the exclusion criteria used, covariates, and the questionnaire-related data acquisition method. The authors are not addressing whether or not patients were on medications for voiding dysfunction including alpha blockers and desmopressin. In Table-1, other covariates, including body mass index, menopausal state, and history of pelvic surgery, are needed to be added because those covariates could impact lower urinary tract symptoms (LUTS) regardless of age. The data acquisition method also must be documented more clearly because the method of data acquisition itself could affect the results of questionnaires, especially in older people with decreased cognitive functioning (2).

Second, there are several additional pitfalls regarding the reporting of the contents of the study, including not dividing the participants by gender and reporting the results of a 3-consecutive-day voiding diary or the Indevus Urgency Severity Scale (IUSS), which the authors clearly mention in the methods section. Considering the fact that pathophysiology of OAB is known to differ according to sex, covariates including prostate size and menopausal state are potent risk factors for aggravation of LUTS in male and female patients, respectively (3, 4), and the authors did not consider such covariates during data analysis.

Lastly, the authors must consider age during correlation analysis. As the authors state in Table-2, age impacts OAB symptoms quite seriously. Partial correlation after adjustment for age would be a far better analytic strategy.

Although the authors demonstrated remarkable results concerning the association between OAB symptom severity and degree of dementia, it is possible that such results are mere associations due to other potent confounding factors. Other potential covariates must be considered and sound statistical analyses using regression analysis after adjustment for age and other potential covariates must be carried out. Considering that the database of this study cohort is hospital-based, acquisition of information regarding other covariates should be possible, and obtaining that information would yield more scientific research on this issue.
